# Supplementary material for: Improving the Enzymatic Activity and Stability of a Lytic Polysaccharide Monooxygenase
Source: Int J Mol Sci. 2023 May 18;24(10):8963. doi: 10.3390/ijms24108963 (PMC10218905; doi:10.3390/ijms24108963)
Supplement: Supplementary file 1 [file ijms-24-08963-s001.zip › ijms-2317245-supplementary.pdf]

Improving the Enzymatic Activity and Stability of a Lytic Polysaccharide Monooxygenase

Miesho Hadush Berhe[1,2,3,4,⊥] Xiangfei Song,[1,2,] and Lishan Yao[1,2,*]

[1]Qingdao New Energy Shandong Laboratory, Qingdao Institute of Bioenergy and Bioprocess Technology, Chinese Academy of Sciences, Qingdao, 266101, China
[2]Shandong Energy Institute, Qingdao, 266101, China
[3]University of Chinese Academy of Sciences, Beijing, 100049, China
[4]Department of Biotechnology, College of Natural and Computational Sciences, Aksum University, 1010, Axum, Ethiopia

Supporting Information

Table S1. Primers used for mutagenesis

**Mutants**	**Sequences (5'--3')**
Y3F-F	TTTATTAAAGAGCCGGTAAGCAGAGC
Y3F-R	TCCGTGACCTCCTATTTGTT CG
I4V-F	TATG TTAAAGAGCCGGTAAGCAGAGCAT
I4V-R	TCCGTGACCTCCTATTTGTT CG
K5E-F	TATATTGAAGAGCCGGTAAGCAGAGCATATAT
K5E-R	TCCGTGACCTCCTATTTGTT CG
N36A-F	GCA CTGAAAGTCAGC GACGGCAG
N36A-R	GATGTGGGTT TCGGGTTTAA CTTCT
N36E-F	GAA CCGCA GAGCGTTGAAGGTC
N36E-R	ATCAATTACT GAACCGTATT TTTGGGCT
V40E-F	GAA GACGGCAGCAGCGAAATCT
V40E-R	ACTTTCAGG TTGATGTGGG TTTC
V40A-F	GCT GAAGGTCCGAAAGGTTTCCC
V40A-R	GCTCTGCGGG TTATCAATTA CTG
V40I-F	ATC GAAGGTCCGAAAGGTTTCCC
V40I-R	GCTCTGCGGG TTATCAATTA CTG
V40L-F	CTG GAAGGTCCGAAAGGTTTCCC
V40L-R	GCTCTGCGGG TTATCAATTA CTG
V40M-F	ATG GAAGGTCCGAAAGGTTTCCC
V40M-R	GCTCTGCGGG TTATCAATTA CTG
R55Q-F	CAG ATAGCA AGCGCAAATGGTG GTAG
R55Q-R	ACCATCCGGG GGACCTG
N60G-F	GGT GGTGGTAGCGGTCAGATTGA
N60G-R	TGCGCTTGCTATACGACCAT C
H77R-F	CGT TGGGTTAAACAGAATATCAGAGG
H77R-R	GTCTGCTGTCTGTTTATCTA AG
E124D-F	GAT CTGATTGGCACCGTTAATCATG
E124D-R	AAATTCGTCA CGGCTCAGCG
G158A-F	GCA GTTTGGGATGTTGCAGATACC
G158A-R	CAGAATGATG TGATAGCCGCT

Table S2. Apparent kinetic parameters of the purified enzymes

Purified enzymes	k_{cat} (min^{-1})	K_m (mM)
V40L-E124D	5.15 ± 0.07	33.53 ± 1.6
I4V-E124D	4.74 ± 0.25	95.46 ± 9.5
V40L-Y3F	3.98 ± 0.05	30.85 ± 1.29
V40L-I4V	4.09 ± 0.27	53.45 ± 9.23
E124D-Y3F	5.02 ± 0.31	93.29 ± 9.16
V40L	5.18 ± 0.12	32.55 ± 3.6
I4V	3.92 ± 0.28	71.31 ± 7.8
E124D	3.76 ± 0.03	13.73 ± 0.82
Y3F	3.24 ± 0.08	38.33 ± 3.12
V40M	3.75 ± 0.05	27.69 ± 1.49
K5E	3.19 ± 0.08	15.59 ± 1.78
V40I	3.18 ± 0.14	25.36 ± 3.84
V40A	2.40 ± 0.09	51.46 ± 5.34
R55Q	2.48 ± 0.01	13.09 ± 0.41
N36E	2.55 ± 0.06	29.02 ± 2.43
WT	2.50 ± 0.27	41.33 ±7.33

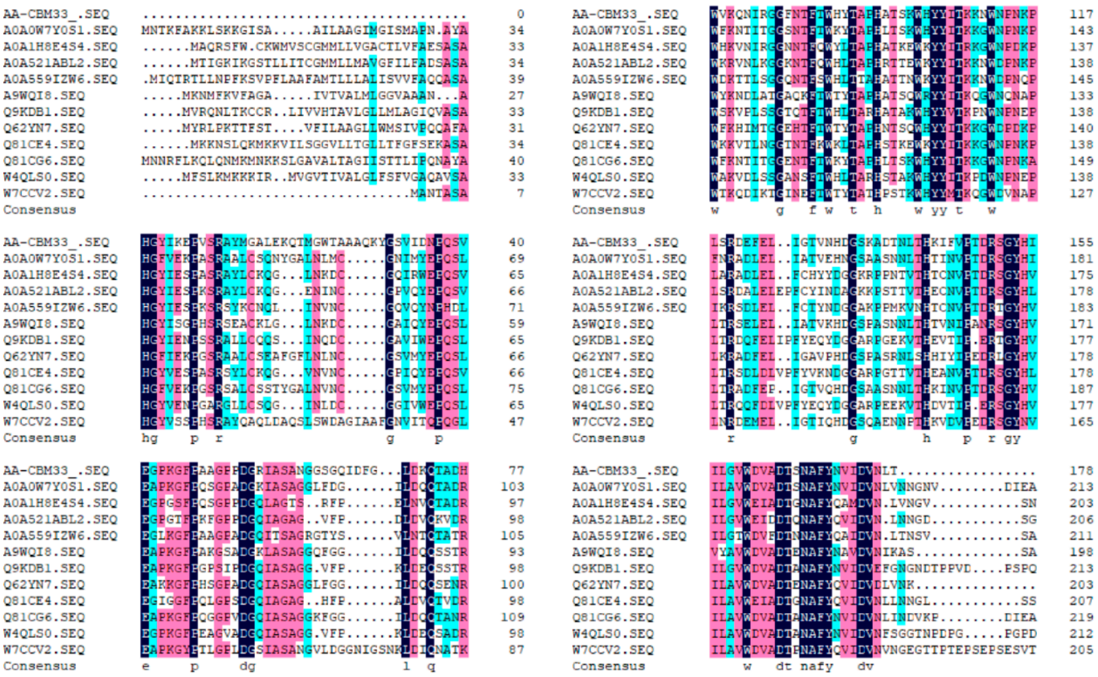

Figure S1. Sequences alignment of *Ba*LPMO10A (AA-CBM33) and its homologs. The multiple amino acid sequence alignment was generated using the DNAMAN software.

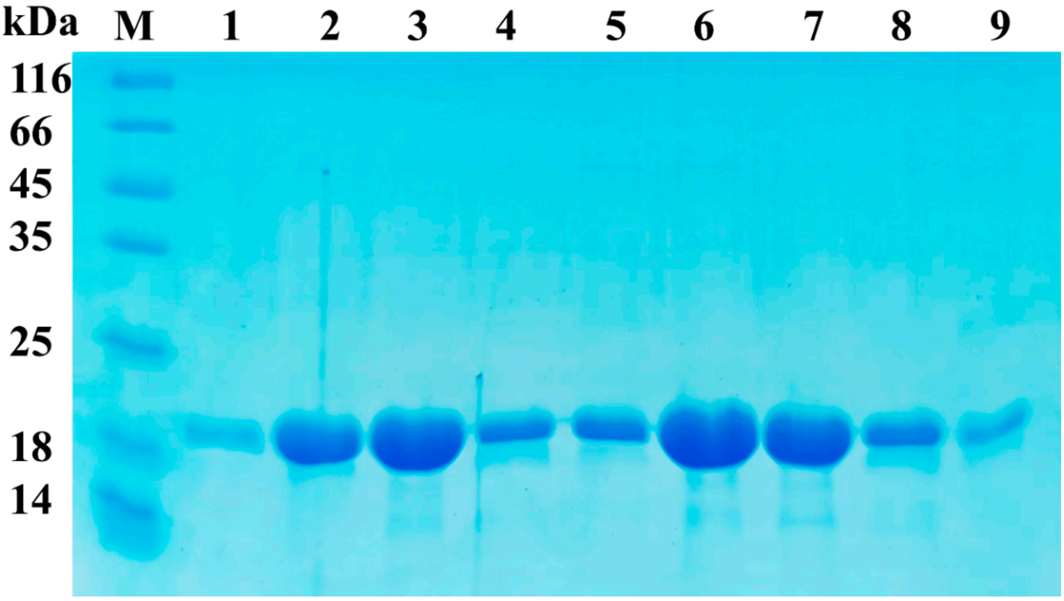

Figure S2. SDS-PAGE of the purified proteins; (M: standard protein marker),the lanes 1, 2, 3, 4, 5, 6, 7, 8, and 9 represents H77R, WT, V40L, N36E, V40I, I4V, E124D, Y3F, and V40M respectively. The remaining mutants have a similar purity. But they were not run on the same SDS-PAGE and thus are not shown.

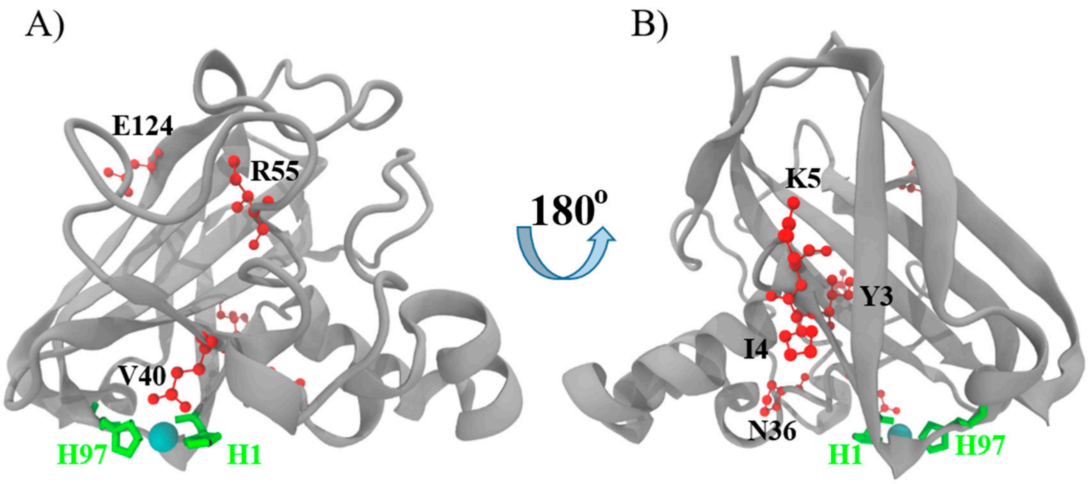

Figure S3. Structural representation of *Ba*LPMO10A (PDB 2YOX), The active site is close to H1, showing copper active site (side chains colored green) and higher activity mutation site (side chains colored red). The distances from the higher activity mutation site's Cα V40, I4, Y3, E124, K5, R55 and N36 to copper ions is 8.0 Å, 15.9 Å, 13.8 Å, 24.2 Å, 19.1 Å, 17.8 Å and 11.5 Å respectively.

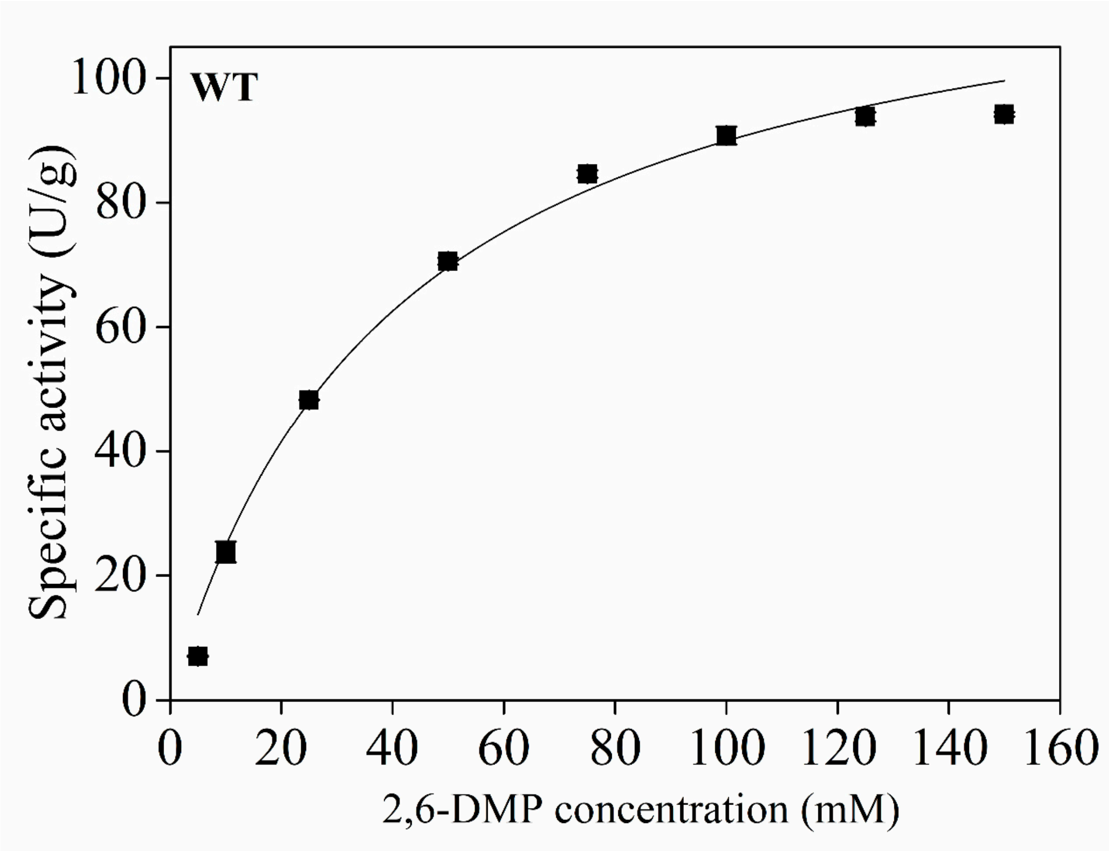

Figure S4: Apparent kinetic parameters of the purified enzyme wild type (WT). It is generated and analyzed by fitting using the OriginPro software.
